# “色谱分离新材料专辑” 引言

**DOI:** 10.3724/SP.J.1123.2023.09005

**Published:** 2023-10-08

**Authors:** Hongdeng QIU, jia CHEN

随着人类科学技术的不断进步,人们面临的分析样品日益复杂,色谱作为复杂样品的重要分离分析技术之一,在化学化工、生命分析、环境监测、材料科学、生物医药、食品安全等诸多领域扮演着越来越重要的角色。色谱分离的核心是色谱柱,色谱柱的灵魂是色谱分离材料。色谱分离的选择性和效率极大程度上取决于所采用的色谱分离材料。因此,色谱分离材料一直是色谱研究领域的前沿和热点之一。

受《色谱》期刊委托,我们组织了“色谱分离新材料”专辑,非常荣幸邀请到国内本领域的十几名专家学者为本专辑撰稿。经过同行专家严格评审,最终出版视角1篇、专论与综述7篇、研究论文4篇,涵盖了液相色谱、气相色谱、毛细管电色谱、超临界流体色谱、芯片毛细管电泳、样品前处理等数个色谱研究领域,内容涉及共价有机框架、金属有机框架、碳点、低共熔溶剂、聚合物、多孔有机笼等多种新材料。

希望通过本专辑为相关研究人员提供帮助,给色谱基础和应用研究同行们带来新的启迪,提升我国色谱分离新材料及相关技术的研究水平。

衷心感谢本专辑作者以及审稿专家的大力支持和倾情奉献!

本专辑客座主编

中国科学院兰州化学物理研究所

邱洪灯 研究员 陈佳 副研究员


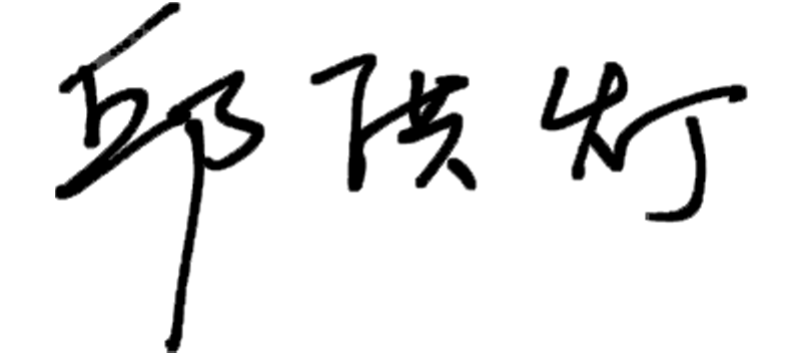
 
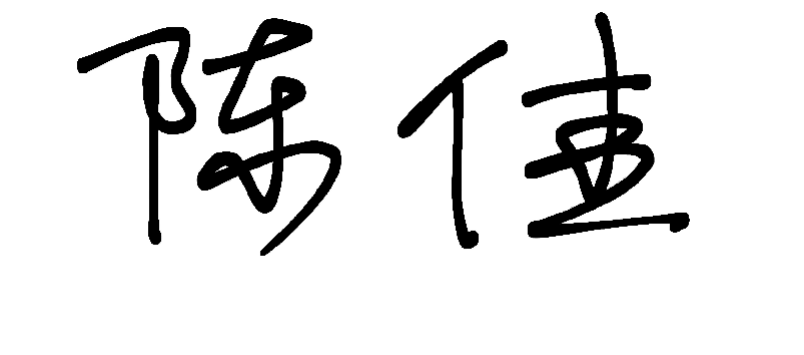


2023年9月3日

